# 1,3-Bis[5-(2-pyrid­yl)-1*H*-tetra­zol-1-yl]propane

**DOI:** 10.1107/S1600536808015274

**Published:** 2008-06-07

**Authors:** Gao-Nan Li, Xiu-Bing Li, Rui-Bin Fang, Qi-Hua Zhao

**Affiliations:** a Department of Chemistry, Key Laboratory of Medicinal Chemistry for Natural Resources, Ministry of Education, Yunnan University, Kunming 650091, People’s Republic of China

## Abstract

The title compound, C_15_H_14_N_10_, is a multidentate ligand obtained by the reaction of 5-(2-pyrid­yl)tetra­zole with 1,3-dibromo­propane. The mol­ecule consists of two 5-(2-pyrid­yl)-1*H*-tetra­zol-1-yl units connected by a propyl­ene bridge in a U-like conformation. A twofold rotation axis passes through the central C atom.

## Related literature

For related literature, see: Bronisz (2002 ▶); Gallardo *et al.* (2004 ▶); Meyer *et al.* (1998 ▶).
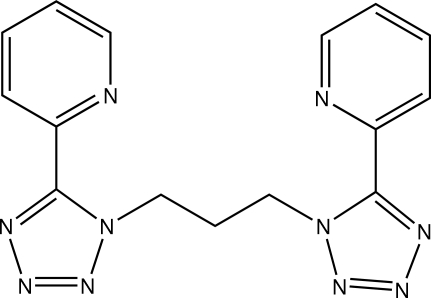


## Experimental

### 

#### Crystal data

C_15_H_14_N_10_
*M* *_r_* = 334.36
Monoclinic, 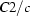

*a* = 14.486 (2) Å
*b* = 9.1322 (13) Å
*c* = 12.8032 (19) Å
β = 111.596 (2)°
*V* = 1574.8 (4) Å^3^
*Z* = 4
Mo *K*α radiation
μ = 0.10 mm^−1^
*T* = 298 (2) K
0.2 × 0.2 × 0.2 mm

#### Data collection

Rigaku Scxmini 1K CCD area-detector diffractometer
Absorption correction: multi-scan (*CrystalClear*; Rigaku, 2005 ▶) *T*_min_ = 0.981, *T*_max_ = 0.981
4930 measured reflections
1845 independent reflections
1166 reflections with *I* > 2σ(*I*)
*R*_int_ = 0.031

#### Refinement

*R*[*F* ^2^ > 2σ(*F* ^2^)] = 0.044
*wR*(*F* ^2^) = 0.112
*S* = 1.02
1845 reflections
115 parameters
H-atom parameters constrained
Δρ_max_ = 0.14 e Å^−3^
Δρ_min_ = −0.14 e Å^−3^

### 

Data collection: *CrystalClear* (Rigaku, 2005 ▶); cell refinement: *CrystalClear*; data reduction: *CrystalClear*; program(s) used to solve structure: *SHELXS97* (Sheldrick, 2008 ▶); program(s) used to refine structure: *SHELXL97* (Sheldrick, 2008 ▶); molecular graphics: *SHELXTL* (Sheldrick, 2008 ▶); software used to prepare material for publication: *SHELXTL*.

## Supplementary Material

Crystal structure: contains datablocks I, global. DOI: 10.1107/S1600536808015274/hg2397sup1.cif

Structure factors: contains datablocks I. DOI: 10.1107/S1600536808015274/hg2397Isup2.hkl

Additional supplementary materials:  crystallographic information; 3D view; checkCIF report

## Figures and Tables

**Fig. 1.**
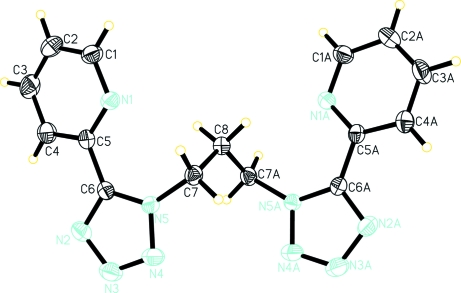
The molecular structure of the title molecule, with the atom-numbering scheme. Displacement ellipsoids are drawn at the 30% probability level (Symmetry code(A): (-x+1,y,-z+1/2)

## supplementary crystallographic information

### Comment

The tetrazolate anion is an ambident system in which alkylation can occur at the N-1 or N-2 position, the relative proportions of which depend upon the reaction conditions, the nature of the alkylating agent and the influence of the 5-substituent (Meyer *et al.*, 1998; Bronisz, 2002). The crystal structure of one of the three regioisomers has already been published (Gallardo *et al.*, 2004). In our case, no regioselectivity was observed and the three possible regioisomers were isolated in equal amounts.

The structure of the title compound (I) is similar to that observed in the paper (Gallardo *et al.*, 2004) with bond lengths and angles in good agreement with expected values. The molecules of (I) are disposed about a crystallographic two-fold axis of symmetry with symmetrical 5-(2-pyridyl)- 2*H*-tetrazolyl units connected by a propylene bridge.  The molecule is folded at the center of the bridge [C7-C8-C7^i^ 116.1 (2)°; symmetry code (i) = (-x+1,y,-z+1/2)] giving a U-like conformation to the free ligand. The inter-ring dihedral angle Py/Tz is 12.00 (7)°.

### Experimental

5-(2-Pyridyl)tetrazole, (3.0 g, 20.0 mmol) was dissolved in 25 ml of 2-butanone with stirring and to the solution 1,3-dibromopropane (2.0 g, 10.0 mmol) and K_2_CO_3_ (5.5 g, 40.0 mmol) were added. The reaction mixture was heated under reflux for 24 h. After cooling the inorganic materials were filtered off and the solvent was removed under reduced pressure to afford the mixture of isomers. These isomers were separated by column chromatography on silica gel (1:4–2:1 EtOAc/Petroleum ether(60-90°C). The pure compound (I) (334 mg, 1.0 mmol) was dissolved in the solvent (1:1 EtOAc/Petroleum ether), and recrystallized from EtOAc/Petroleum ether affording colorless crystals. .

### Refinement

Positional parameters of all H atoms were calculated geometrically and were allowed to ride on the C atoms to which they are bonded, with C-H distances in the range 0.93-0.97Å and *U*_iso_(H) = 1.2*U*_eq_(C).

### Crystal data

**Table:**

C_15_H_14_N_10_	*F*_000_ = 696
*M**_r_* = 334.36	*D*_x_ = 1.410 Mg m^−^^3^
Monoclinic, *C*2/*c*	Mo *K*α radiation λ = 0.71073 Å
Hall symbol: -C 2yc	Cell parameters from 25 reflections
*a* = 14.486 (2) Å	θ = 2.7–28.3º
*b* = 9.1322 (13) Å	µ = 0.10 mm^−^^1^
*c* = 12.8032 (19) Å	*T* = 298 (2) K
β = 111.596 (2)º	Block, colourless
*V* = 1574.8 (4) Å^3^	0.2 × 0.2 × 0.2 mm
*Z* = 4	

### Data collection

**Table:**

Rigaku Scxmini 1K CCD area-detector diffractometer	1845 independent reflections
Radiation source: fine-focus sealed tube	1166 reflections with *I* > 2σ(*I*)
Monochromator: graphite	*R*_int_ = 0.031
Detector resolution: 8.192 pixels mm^-1^	θ_max_ = 28.3º
*T* = 298(2) K	θ_min_ = 2.7º
thin–slice ω scans	*h* = −15→18
Absorption correction: multi-scan(CrystalClear; Rigaku, 2005)	*k* = −7→12
*T*_min_ = 0.981, *T*_max_ = 0.981	*l* = −16→16
4930 measured reflections	

### Refinement

**Table:**

Refinement on *F*^2^	Hydrogen site location: inferred from neighbouring sites
Least-squares matrix: full	H-atom parameters constrained
*R*[*F*^2^ > 2σ(*F*^2^)] = 0.044	*w* = 1/[σ^2^(*F*_o_^2^) + (0.0498*P*)^2^ + 0.113*P*] where *P* = (*F*_o_^2^ + 2*F*_c_^2^)/3
*wR*(*F*^2^) = 0.112	(Δ/σ)_max_ < 0.001
*S* = 1.02	Δρ_max_ = 0.14 e Å^−^^3^
1845 reflections	Δρ_min_ = −0.14 e Å^−^^3^
115 parameters	Extinction correction: SHELXL97 (Sheldrick, 2008), Fc^*^=kFc[1+0.001xFc^2^λ^3^/sin(2θ)]^-1/4^
Primary atom site location: structure-invariant direct methods	Extinction coefficient: 0.0018 (5)
Secondary atom site location: difference Fourier map	

### Special details

**Table:**

Geometry. All e.s.d.'s (except the e.s.d. in the dihedral angle between two l.s. planes) are estimated using the full covariance matrix. The cell e.s.d.'s are taken into account individually in the estimation of e.s.d.'s in distances, angles and torsion angles; correlations between e.s.d.'s in cell parameters are only used when they are defined by crystal symmetry. An approximate (isotropic) treatment of cell e.s.d.'s is used for estimating e.s.d.'s involving l.s. planes.
Refinement. Refinement of *F*^2^ against ALL reflections. The weighted *R*-factor *wR* and goodness of fit *S* are based on *F*^2^, conventional *R*-factors *R* are based on *F*, with *F* set to zero for negative *F*^2^. The threshold expression of *F*^2^ > σ(*F*^2^) is used only for calculating *R*-factors(gt) *etc*. and is not relevant to the choice of reflections for refinement. *R*-factors based on *F*^2^ are statistically about twice as large as those based on *F*, and *R*- factors based on ALL data will be even larger.

### Fractional atomic coordinates and isotropic or equivalent isotropic displacement parameters (Å^2^)

**Table:**

	*x*	*y*	*z*	*U*_iso_*/*U*_eq_	Occ. (<1)
C2	0.37119 (12)	0.2927 (2)	−0.15496 (13)	0.0542 (5)	
H2A	0.3811	0.2170	−0.1982	0.065*	
N2	0.39676 (9)	0.07129 (14)	0.09971 (10)	0.0372 (3)	
N3	0.40148 (11)	−0.07629 (15)	0.10268 (13)	0.0520 (4)	
N4	0.38893 (11)	−0.11910 (16)	0.00162 (14)	0.0592 (4)	
N5	0.37593 (11)	−0.00222 (17)	−0.06788 (12)	0.0527 (4)	
C1	0.36961 (10)	0.26606 (18)	−0.04949 (12)	0.0388 (4)	
N1	0.35696 (10)	0.37150 (15)	0.01654 (10)	0.0447 (4)	
C3	0.34557 (13)	0.50772 (19)	−0.02391 (15)	0.0517 (5)	
H3B	0.3380	0.5826	0.0215	0.062*	
C4	0.34441 (14)	0.5440 (2)	−0.12819 (15)	0.0579 (5)	
H4B	0.3349	0.6404	−0.1533	0.069*	
C5	0.35767 (15)	0.4344 (2)	−0.19443 (15)	0.0642 (6)	
H5B	0.3575	0.4556	−0.2655	0.077*	
C6	0.38094 (11)	0.11518 (17)	−0.00589 (12)	0.0382 (4)	
C7	0.40529 (11)	0.15178 (18)	0.20192 (12)	0.0411 (4)	
H7A	0.4022	0.0827	0.2581	0.049*	
H7B	0.3491	0.2177	0.1851	0.049*	
C8	0.5000	0.2392 (2)	0.2500	0.0418 (5)	
H8B	0.4950	0.3021	0.3087	0.063*	0.50
H8A	0.5050	0.3021	0.1913	0.063*	0.50

### Atomic displacement parameters (Å^2^)

**Table:**

	*U*^11^	*U*^22^	*U*^33^	*U*^12^	*U*^13^	*U*^23^
C2	0.0685 (12)	0.0581 (13)	0.0403 (9)	0.0009 (9)	0.0250 (8)	−0.0008 (9)
N2	0.0384 (7)	0.0343 (8)	0.0384 (7)	−0.0012 (6)	0.0136 (5)	0.0013 (6)
N3	0.0556 (9)	0.0364 (9)	0.0627 (10)	−0.0013 (7)	0.0203 (7)	0.0003 (7)
N4	0.0665 (10)	0.0428 (9)	0.0669 (11)	−0.0018 (7)	0.0228 (8)	−0.0092 (8)
N5	0.0594 (9)	0.0484 (9)	0.0490 (9)	−0.0014 (7)	0.0188 (7)	−0.0109 (7)
C1	0.0354 (8)	0.0455 (10)	0.0342 (8)	−0.0019 (7)	0.0112 (6)	−0.0013 (7)
N1	0.0554 (8)	0.0395 (8)	0.0404 (8)	0.0038 (6)	0.0192 (6)	0.0028 (6)
C3	0.0619 (11)	0.0418 (10)	0.0533 (10)	0.0044 (8)	0.0234 (9)	0.0059 (9)
C4	0.0646 (12)	0.0524 (12)	0.0590 (11)	0.0033 (9)	0.0256 (9)	0.0179 (10)
C5	0.0799 (14)	0.0731 (15)	0.0431 (10)	−0.0029 (11)	0.0269 (9)	0.0142 (10)
C6	0.0369 (8)	0.0412 (9)	0.0353 (8)	−0.0003 (7)	0.0119 (6)	−0.0043 (7)
C7	0.0465 (9)	0.0453 (10)	0.0342 (8)	0.0013 (7)	0.0180 (7)	0.0021 (7)
C8	0.0472 (13)	0.0396 (13)	0.0357 (11)	0.000	0.0120 (9)	0.000

### Geometric parameters (Å, °)

**Table:**

C2—C5	1.377 (2)	C3—C4	1.370 (2)
C2—C1	1.381 (2)	C3—H3B	0.9300
C2—H2A	0.9300	C4—C5	1.370 (3)
N2—N3	1.3493 (18)	C4—H4B	0.9300
N2—C6	1.3464 (18)	C5—H5B	0.9300
N2—C7	1.4661 (19)	C7—C8	1.5091 (18)
N3—N4	1.2988 (19)	C7—H7A	0.9700
N4—N5	1.358 (2)	C7—H7B	0.9700
N5—C6	1.3199 (19)	C8—C7^i^	1.5091 (18)
C1—N1	1.3378 (19)	C8—H8B	0.9700
C1—C6	1.473 (2)	C8—H8A	0.9700
N1—C3	1.334 (2)		
			
C5—C2—C1	118.27 (16)	C4—C5—C2	119.51 (16)
C5—C2—H2A	120.9	C4—C5—H5B	120.2
C1—C2—H2A	120.9	C2—C5—H5B	120.2
N3—N2—C6	108.29 (13)	N5—C6—N2	108.26 (14)
N3—N2—C7	119.27 (12)	N5—C6—C1	123.92 (14)
C6—N2—C7	132.41 (13)	N2—C6—C1	127.81 (14)
N4—N3—N2	106.59 (13)	N2—C7—C8	113.25 (11)
N3—N4—N5	110.55 (14)	N2—C7—H7A	108.9
C6—N5—N4	106.31 (13)	C8—C7—H7A	108.9
N1—C1—C2	123.13 (15)	N2—C7—H7B	108.9
N1—C1—C6	117.12 (13)	C8—C7—H7B	108.9
C2—C1—C6	119.75 (15)	H7A—C7—H7B	107.7
C3—N1—C1	116.87 (14)	C7—C8—C7^i^	116.09 (19)
N1—C3—C4	124.01 (17)	C7—C8—H8B	108.3
N1—C3—H3B	118.0	C7^i^—C8—H8B	108.3
C4—C3—H3B	118.0	C7—C8—H8A	108.3
C5—C4—C3	118.19 (17)	C7^i^—C8—H8A	108.3
C5—C4—H4B	120.9	H8B—C8—H8A	107.4
C3—C4—H4B	120.9		
			
C6—N2—N3—N4	0.07 (16)	N4—N5—C6—C1	−179.43 (14)
C7—N2—N3—N4	178.05 (12)	N3—N2—C6—N5	−0.06 (16)
N2—N3—N4—N5	−0.06 (17)	C7—N2—C6—N5	−177.67 (14)
N3—N4—N5—C6	0.02 (18)	N3—N2—C6—C1	179.36 (14)
C5—C2—C1—N1	−0.9 (2)	C7—N2—C6—C1	1.8 (2)
C5—C2—C1—C6	178.24 (15)	N1—C1—C6—N5	167.21 (14)
C2—C1—N1—C3	0.0 (2)	C2—C1—C6—N5	−12.0 (2)
C6—C1—N1—C3	−179.23 (13)	N1—C1—C6—N2	−12.1 (2)
C1—N1—C3—C4	1.2 (2)	C2—C1—C6—N2	168.64 (14)
N1—C3—C4—C5	−1.3 (3)	N3—N2—C7—C8	111.61 (15)
C3—C4—C5—C2	0.3 (3)	C6—N2—C7—C8	−71.0 (2)
C1—C2—C5—C4	0.8 (3)	N2—C7—C8—C7^i^	−67.96 (10)
N4—N5—C6—N2	0.02 (17)		

Symmetry codes: (i) −*x*+1, *y*, −*z*+1/2.
